# Acceptance of a robotic system for nursing care: a cross-sectional survey with professional nurses, care recipients and relatives

**DOI:** 10.1186/s12912-024-01849-5

**Published:** 2024-03-14

**Authors:** Murielle Madi, Svenja Nielsen, Mona Schweitzer, Maximilian Siebert, Daniel Körner, Sina Langensiepen, Astrid Stephan, Gabriele Meyer

**Affiliations:** 1 Institute of Health and Nursing Science, Medical Faculty, Martin Luther University Halle-Wittenberg, Magdeburger Straße 8, 06112 Halle (Saale), Germany; 2 Department of Nursing Science, Uniklinik RWTH Aachen, Pauwelsstraße 30, 52074 Aachen, Germany; 3 Institute of Applied Medical Engineering, Uniklinik RWTH Aachen, Pauwelsstraße 30, 52074 Aachen, Germany

**Keywords:** Robotics, Nursing, Acceptance, Ethical analysis, Hospitals, Nursing homes, User-centered design, Survey, Evaluation

## Abstract

**Background:**

The end-users’ acceptance is a core concept in the development, implementation and evaluation of new systems like robotic systems in daily nursing practice. So far, studies have shown various findings concerning the acceptance of systems that are intended to assist people with support or care needs. Not much has been reported on the acceptance of robots that provide direct physical assistance to nurses in bedside care. Therefore, this study aimed to investigate the acceptance along with ethical implications of the prototype of an assistive robotic arm aiming to support nurses in bedside care, from the perspective of nurses, care recipients and their relatives.

**Methods:**

A cross-sectional survey design was applied at an early stage in the technological development of the system. Professional nurses, care recipients and relatives were recruited from a university hospital and a nursing home in Germany. The questionnaire was handed out following either a video or a live demonstration of the lab prototype and a subsequent one-to-one follow-up discussion. Data analysis was performed descriptively.

**Results:**

A total of 67 participants took part in the study. The rejection of specified ethical concerns across all the respondents was 77%. For items related to both perceived usefulness and intention to use, 75% of ratings across all the respondents were positive. In the follow-up discussions, the participants showed interest and openness toward the prototype, although there were varying opinions on aspects such as size, appearance, velocity, and potential impact on workload.

**Conclusions:**

Regarding the current state of development, the acceptance among the participants was high, and ethical concerns were relatively minor. Moving forward, it would be beneficial to explore the acceptance in further developmental stages of the system, particularly when the usability is tested.

**Supplementary Information:**

The online version contains supplementary material available at 10.1186/s12912-024-01849-5.

## Background

In healthcare, there is a growing interest in the introduction of technological systems such as robotics [1]. Robotic systems might offer potential physical and mental relief to caregivers and increase the safety as well as the mobility and independence of care recipients [2–6]. It has been suggested that robotic assistance is likely to mitigate the shortage of nurses in the healthcare system by providing support for automated and routine nursing tasks [2]. In 1985, the first robot used in healthcare was introduced, which was an assistive device for neurosurgical biopsies [7]. Nowadays, robotics have been reported to assist in many areas of application such as surgery, administering medication, monitoring of care recipients and hygiene care [8].

The end-users’ acceptance is a core concept in the adoption, implementation and evaluation of new systems in daily healthcare and nursing practice [9, 10]. Therefore, potential end-users such as caregivers and care recipients should be involved in all stages of the system’s developmental processes [11–14]. In the context of technology, acceptance is defined as the “willingness, intention and internal motivation to use a technology” [15]. The Technology Acceptance Model is one approach that aims to explain factors that predict the actual system’s use [16]. It was originally introduced with the components of perceived usefulness, perceived ease of use and intention to use and was further developed several times with growing complexity. The model has been widely validated across different contexts including healthcare and often serves as a basis for evaluating the acceptance of assistive systems. Hence the terms “perceived usefulness”, “ease of use” and “intention to use” are often encountered in studies concerning technology acceptance.

Acceptance research among older adults suggests that they seem to be more open to robotic support in service tasks than in social companionship [17–19]. Service tasks may include delivery services or cleaning, whereas social robots focus more on interaction and communication [18, 20, 21]. However, after encountering social robots in video demonstrations, live demonstrations or testing, older persons showed a predominantly positive attitude towards these robots [22–26]. Service robots are perceived differently. Studies about the acceptance of robots by older people after testing them revealed divergent results. In a study by Cavallo et al. (2018), older people showed a high overall acceptance [27], whereas other studies revealed low intentions to use the applied robot [28, 29].

The perspective of relatives and informal caregivers is rarely considered in acceptance studies concerning robotics in healthcare. Results from qualitative and mixed methods studies revealed conflicting results regarding an assistive robot for older adults. Informal caregivers are open to the idea [30] even more so than people in need of care [28]. But on the other hand, informal caregivers seem to be more skeptical about a social robot when this is compared with professional caregivers and older people [26].

Studies with healthcare professionals and nursing students generally revealed positive attitudes towards assistive robots in professional healthcare settings, including ratings of perceived usefulness and ease of use [2, 31]. In a survey with 576 professional nurses in Germany, the more the participants reported knowledge and confidence in robotics, the more positive and useful they rated robotics and their attitudes towards robotics [5].

In Finland and Japan, research with homecare professionals showed that the Finnish homecare nursing professionals rated the usefulness of care robots lower than the Japanese did and specifically denied the usefulness of care robots in relieving anxiety or loneliness [3, 32]. Therefore, the cultural background determines the acceptance of care robots.

Ethical concerns, alongside cultural background and familiarity with robotics, turned out to be crucial for the acceptance of assistive technology in nursing care. When asked about the challenges related to healthcare robotics, participants expressed their concern regarding the ambiguity surrounding the liability of the robot’s actions [1]. Moreover, since caring is considered the essence of nursing, an ethical concern raised in a study about the ethics of caring is that robots cannot deliver the same care as human carers do. The human touch in nursing, for instance, is unlikely to be replaced by robots [33, 34]. In addition, data protection issues have been raised as an ethical concern that needs to be addressed in a study about the nurses’ view of the use of robots in pediatrics care [35].

Different results have issued from the studies about technology acceptance in healthcare. The variance in results might be due to the difference in cultures, robotic types and each study’s conditions. Not much has been reported on robots that provide direct physical assistance to nurses in bedside care. Furthermore, the perspective of family members of the care recipients regarding technology advancement in healthcare has also found little consideration in the literature.

Therefore, we aimed to investigate the acceptance along with ethical implications of a robotic laboratory prototype (lab prototype) to assist nurses in bedside care, namely from the perspective of nurses, care recipients and their relatives.

## Method

### Study design

A cross-sectional survey study design was applied.

### Sample and setting

The participants were sampled by convenience and recruited from a university hospital and a nursing home in North Rhine-Westphalia, Germany. Professional nurses and members of the research team approached nurses, care recipients and relatives in person, via email and by posting on the internal website of the university hospital. Eligible participants were informed about the study project and asked whether they were interested in participating in the study; if so, they should contact the research team via email. We intended to have a sample size of 20 care recipients, 20 relatives and 30 professional nurses. The intended sample sizes were selected for reasons of feasibility and respect for the capacities of professional nurses, care recipients and relatives in the context of the ever-present staff shortage and exceptional pandemic conditions. Participants were eligible if they.

- were currently care recipients or had been care recipients in the preceding 12 months (in the following: care recipients).
- *or* they were associated with a person in current need of care or with a person who had been in need of care in the preceding 12 months (in the following: relatives).
- *or* were skilled nurses with either vocational or academic training (in the following: professional nurses).

All the participants had to have sufficient cognitive and sensory skills and to be able to speak the German language.

### The assistive system “PfleKoRo”

**Fig. 1:**
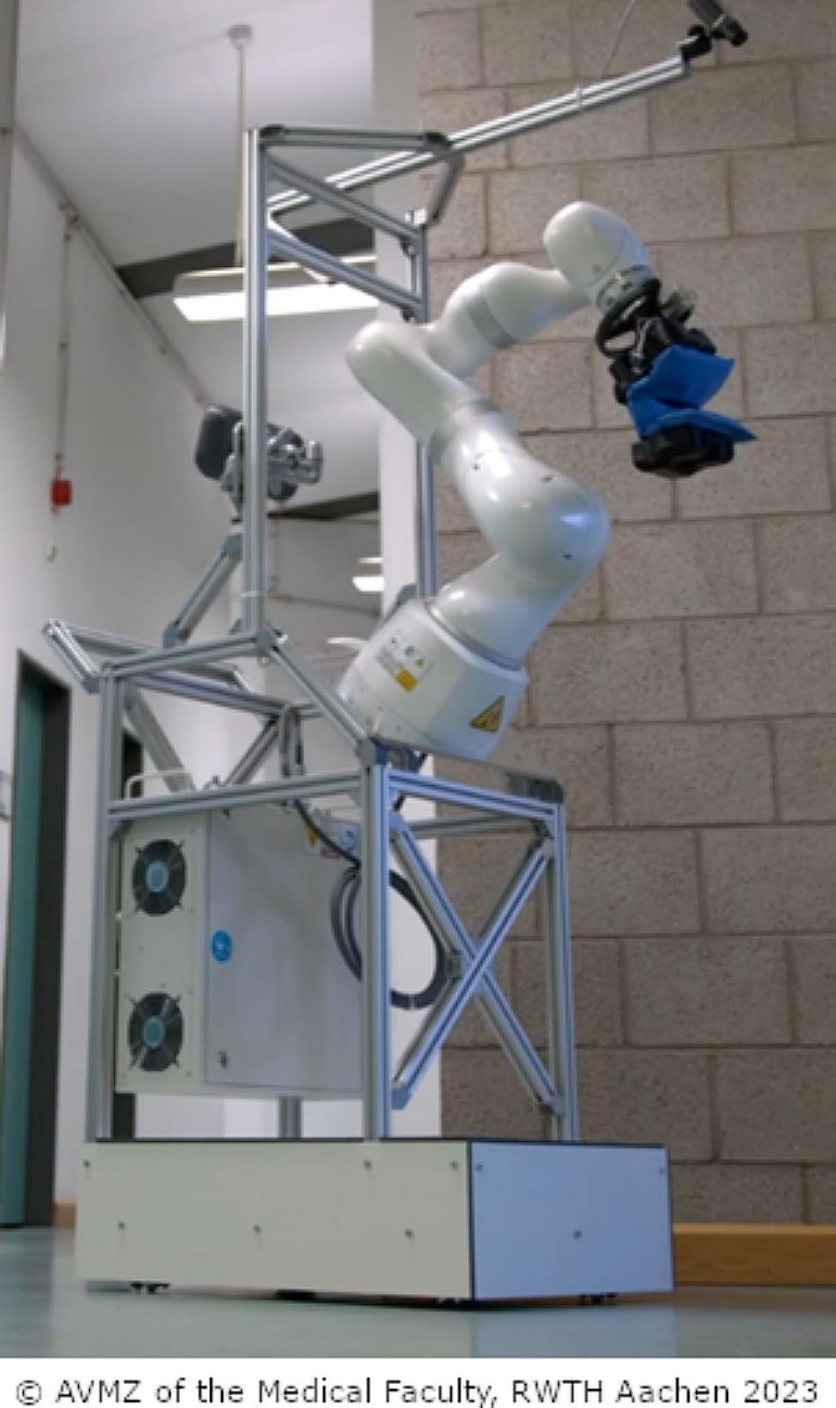
Overall PfleKoRo system

**Fig. 2:**
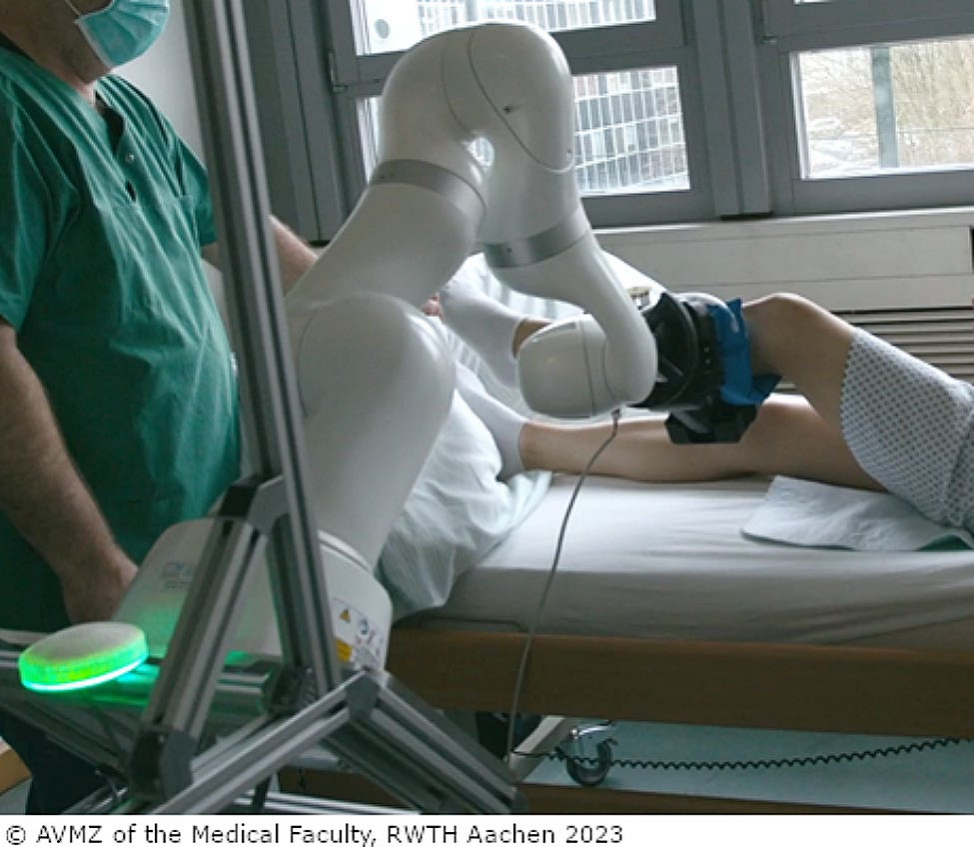
PfleKoRo system in use

The assistive system, which is the object of this study, was based on a seven-axis lightweight robot arm and was intended to be developed to assist nurses when repositioning highly care-dependent and bedridden people and turning them to the side, or holding and lifting their limbs to perform a nursing procedure. For this purpose, the system can be moved to the patient’s bedside and connected to the appropriate contact surface or surfaces for the respective nursing procedure. These intended scenarios were selected according to a user-driven needs assessment earlier in this project [36]. At the time of the study, the laboratory prototype (lab prototype) shown to the participants was in the early stages of development and therefore could not yet be tested by nurses. It also looked rather technical. For an impression of the lab prototype, see Figs. 1 and 2.

### Procedure

**Fig. 3:**
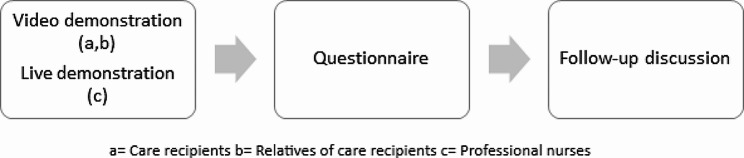
Study procedure

As a basis for evaluation, the questionnaire was preceded by a video or live demonstration, depending on the participant group (see Fig. 3). A follow-up discussion with broad open-ended questions aimed to clarify any confusion related to the lab prototype’s demonstration and to the distributed questionnaire. The questions served as a supplement to the information obtained from the questionnaire. The guides for the follow-up discussion can be found in the additional files 1–3. The care situation that was asked about in the questionnaires refers to the activities performed by the lab prototype in the video and live demonstrations.

In March 2022, members of the project team approached *care recipients and relatives of care recipients* in person at the university hospital and nursing home in face-to-face one-to-one situations. First, a video was shared with the participants that showed the lab prototype assisting a nurse in changing a leg bandage on a healthy person playing the role of a bedridden care recipient. Afterwards, care recipients and relatives were asked to fill in the questionnaire themselves or with assistance from the member of the project team. Subsequently, participants were asked the broad open-ended questions in the follow-up discussion. A member of the research team led the discussion with one participant. As this study focuses on the quantitative results, the follow-up discussions were not recorded, but minutes were taken. For care recipients and relatives, the participation in the study took maximum one hour.

In March and April 2023, the *professional nurses* were invited to the laboratory, where the prototype was being developed. The professional nurses first saw a live demonstration of a nursing situation with support from the lab prototype (lifting and holding a leg; turning care recipient to side). The steps that followed the live demonstration were identical with those described for the care recipients and relatives. The procedure with the professional nurses took a maximum of 90 min. At the end of each session, each participant received a 10€ voucher as compensation for their participation. The participants had no prior knowledge of the voucher before they finished answering the interview questions.

### Questionnaire

The questionnaire was created exclusively for this study and consisted of two main sections. One section was related to ethical implications and the other to perceived usefulness/ intention of use with up to 24 statements, depending on the study population. The ethical implication statements in Sect. 1 were formulated on the basis of a literature search and individual and focus group interviews in earlier phases of the project [35]. Section 2 concerned the perceived usefulness/ intention of use and was based on the Technology Acceptance Model, adapted to the project and study population [16]. The participants were asked to indicate their level of agreement with the statements on a 5-point-Likert scale from “do not agree at all” to “strongly agree”. One further section included questions about general participant information. The questionnaires can be found in the additional files 4–6.

The survey was pretested with two professional nurses who were members of the research team and supported the researchers throughout the project. A nurse researcher asked the professional nurses how they understood each question and how they would answer it and then compared their replies with a set of references. The set of references included the meaning of each question as perceived by the members of the research team who had developed the survey, and were based on definitions from the TAM [16]. All the involved team members tested the entire procedure including the demonstration of the lab prototype, the questionnaire and follow-up discussion and made improvements iteratively before the actual data collection with the study participants took place. Separate training sessions were held with the team members who were involved in the data collection for the live demonstration and the follow-up discussion.

### Ethical considerations

The study was approved by the ethics committee of the Medical Faculty of the Rheinisch-Westfälische Technische University Aachen (462 − 21; 416 − 22). The official employee representatives at the university hospital also gave written approval to conduct the study. The participation was voluntary, and a written informed consent was obtained from each participant before inclusion in the study. All survey data were processed and stored anonymously and were accessible for team members only. The questionnaire and notice sheets were marked only with numbers.

### Data analysis

Data from the questionnaires were analyzed descriptively using Microsoft Forms and Microsoft Excel. For the follow-up discussions, all the notes were collected and grouped according to the question in Microsoft Excel and in addition structured thematically on a digital whiteboard.

## Results

### Descriptive characteristics of participants

Eighty people showed their interest in participating in the study. In the end, eight did not participate due to health problems and five did not participate due to a shortage of nurses on the ward. A total of 67 people participated in the study, including 27 professional nurses, 20 care recipients and 20 relatives. The majority was female (67.2%). The participants’ age group ranged between 20 and 81 years. One participant over 60 years was a professional nurse while all others over 60 years were care recipients and relatives. Twenty-eight participants were recruited from a nursing home and 39 from a university hospital. On a Likert scale from 1 to 5, the participants rated themselves as more technically confident than skeptical with an average of 3.85 points and with only marginal differences between groups. Table 1 displays the participants’ characteristics (see additional file 7 for the participants’ characteristics per group).

**Table 1:** Characteristics of participants

Characteristics		*n* = 67
Group of participants	Nurses	27 (40.3)
	Care recipients	20 (29.9)
	Relatives	20 (29.9)
Gender	Female	45 (67.2)
	Male	22 (32.8)
Age, years	20–30	15 (22.4)
	31–40	11 (16.4)
	41–50	9 (13.4)
	51–60	13 (19.4)
	> 60	20 (29.9)
Setting	Nursing home	28 (41.8)
	University hospital	39 (58.2)
Mean technological confidence, Likert scale 1–5 [SD]		3.85 [1.05]

Values are numbers (percentage), unless stated otherwise.

### Questionnaire

**Fig. 4:**
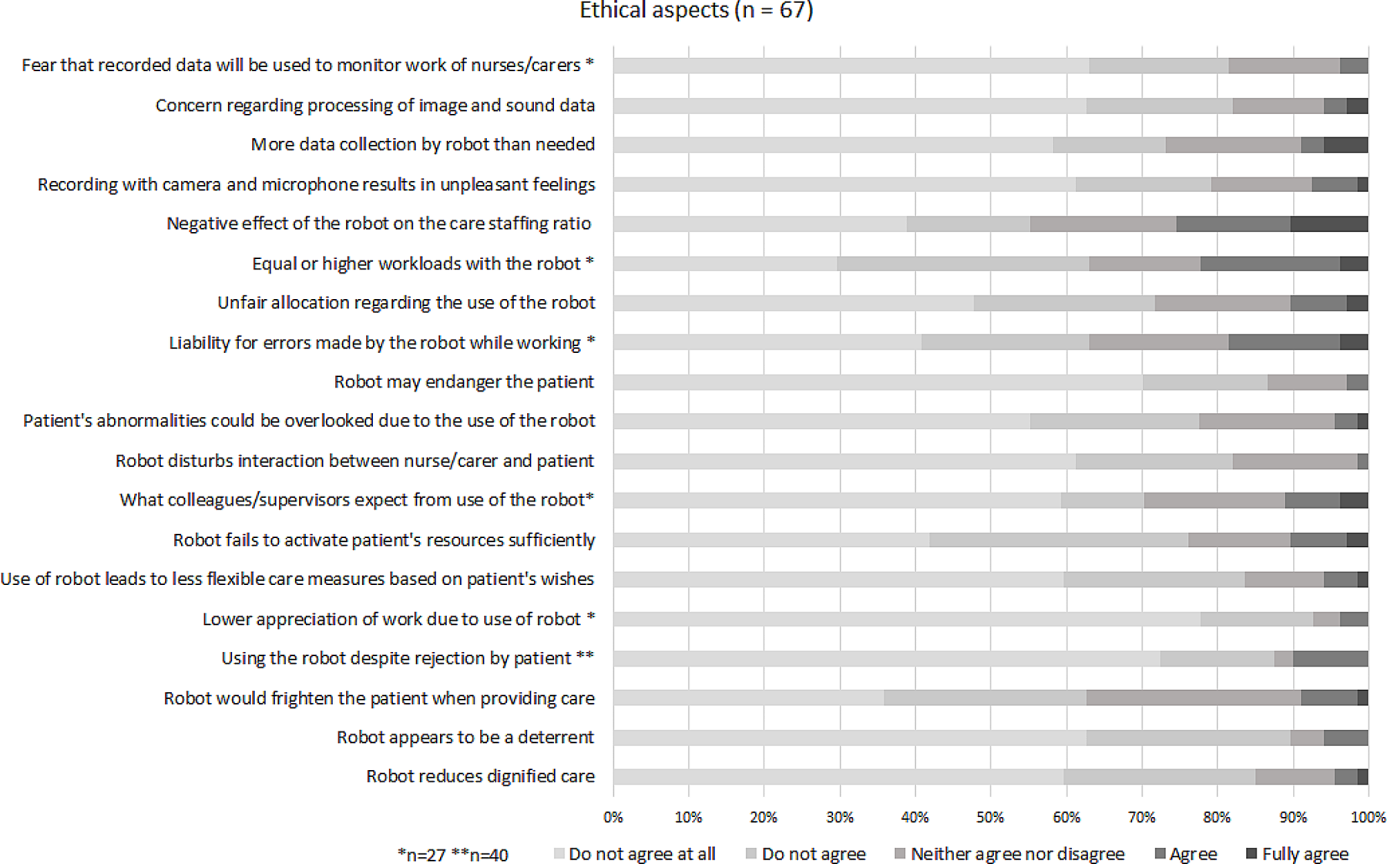
Results on ethical aspects (all respondent groups)

**Fig. 5:**
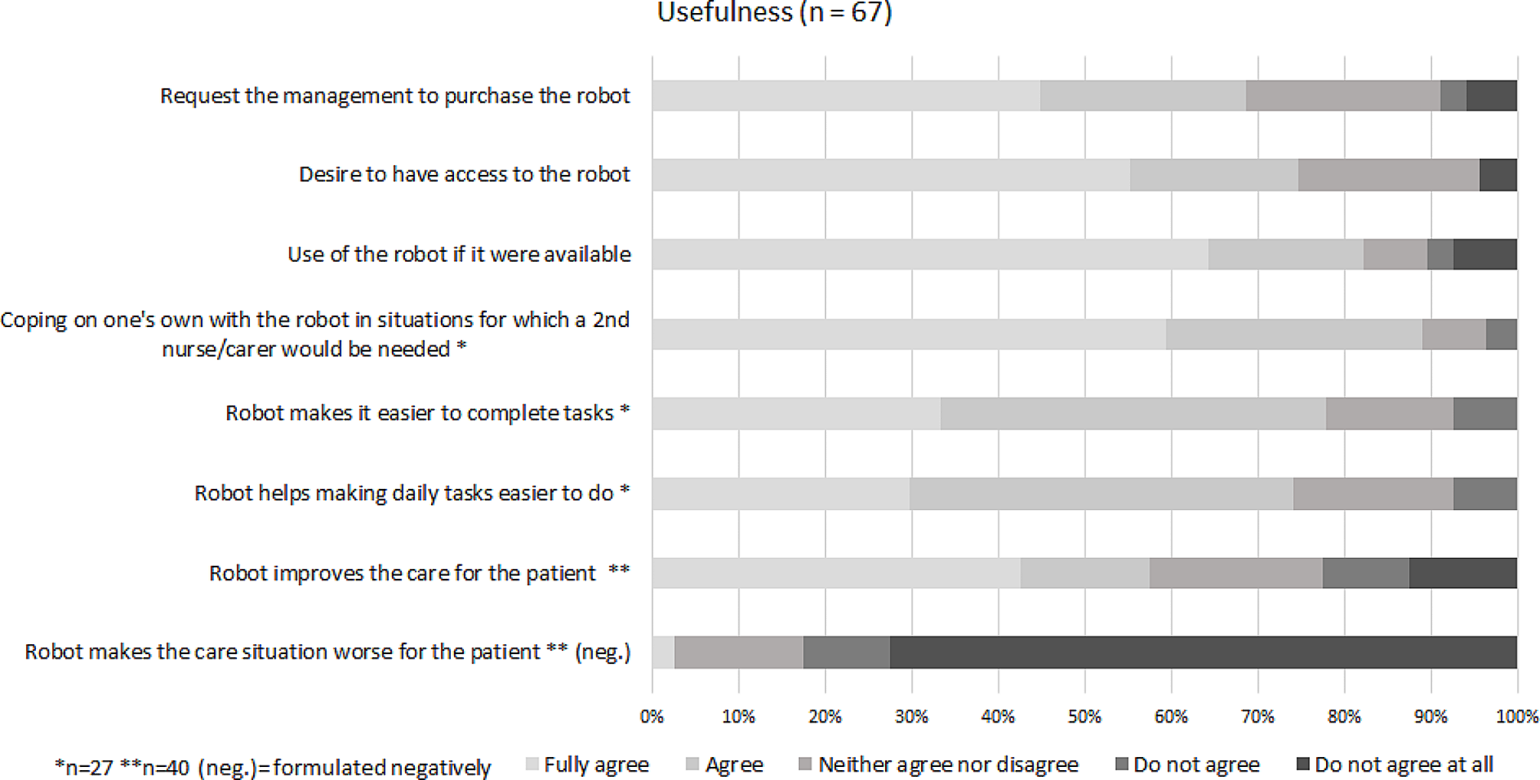
Results on usefulness (all respondent groups)

The results of the questionnaire throughout all of the respondent groups are presented in Figs. 4 and 5. The results for each group can be viewed in the additional files 8–10.

#### Ethical aspects

In the questionnaires, there was a low agreement on ethical concerns in all groups of participants. The overall agreement with ethical concerns across all items and respondents was 7%. A total of 77% of items representing ethical concerns were rejected by the participants. For example, a large proportion of 90% stated that they do not find the system to be deterrent. 93% of the participating nurses negated that using the system could lead to a lower appreciation of their work and 85% of all respondents did not fear that the robot would reduce dignified care. Another 82% did not fear the processing of image and sound data through the system’s camera and microphone. Furthermore, 86% of the respondents were not afraid that the care recipients would be endangered and 82% disagreed that the interaction between the professional nurse and the care recipient would be disrupted by using the robot.

Participants distinctly confirmed the fear that the system could have a negative impact on the staff ratio. A total of 25% agreed with this statement, 23% were indecisive. Moreover, when asked if the system would frighten care recipients, almost a third (28%) of professional nurses and relatives were indecisive, whereas 8% agreed. However, the participating care recipients themselves did not consider the system to be frightening. Likewise, none of the participating care recipients feared the system could cause them injuries.

Apart from concerns about the staffing ratio, every single ethical concern was confirmed by two out of 20 care recipients at most, and by a maximum of two out of 20 relatives. Among the professional nurses, there was a slightly stronger agreement to ethical concerns. Concerns about the staffing ratio and intimidation of care recipients aside, 23% of the professional nurses confirmed they feared the workload would be the same or higher with the system than without it, while another 15% were indecisive. A total of 19% of professional nurses were also in agreement with concerns about both liability and an unfair allocation of the system.

#### Perceived usefulness and intention to use

For items related to both perceived usefulness and intention to use, 75% of ratings across all respondents were positive with 4/5 or 5/5 points. The participants rated 10% of perceived usefulness items and 8% of intention to use items negatively with 1/5 or 2/5 points.

Among the care recipients and relatives, 58% agreed that the lab prototype could improve the care situation for a care recipient, while 23% disagreed with this statement. At the same time, 3% of care recipients and relatives agreed with the opposite statement, stating that the lab prototype made the care situation worse for the care recipient, while 83% disagreed with that.

In the group of professional nurses, 89% agreed they could cope on their own with the lab prototype in situations where a second nurse is usually required. A total of 77% of the nurses agreed that the lab prototype made it easier to complete tasks. However, 7% of the professional nurses disagreed with both statements.

Across all the participants, 82% indicated that they would use or agree to the use of the lab prototype if it were available. Agreement with this statement was slightly higher among nurses (85% agreement) than among care recipients and relatives (75% agreement each). Similarly, 69% of all the respondents stated that they would request management to purchase the lab prototype, with the highest agreement being among the nurses (75%) and the lowest agreement among the care recipients (60%).

### Follow-up discussion

The first reactions in the follow-up discussion were mostly positive. The participants were interested, fascinated and open-minded. One relative said that this is the future of nursing care: cooperation between humans and machines (Relative (R) 22). A person in need of care said that the system could provide support that sustains the professional nurse and enables more time and attention for care recipients such as himself (Care recipient 17).

However, a few initial reactions were fundamentally skeptical. A relative found that it was “not a good thing”, and nursing care should only be done by humans without machines (R 21). A professional nurse expressed the opinion that other problems in nursing care were more important than developing a robotic system (Nurse N 10). Both the relative and the professional nurse wished for more staff instead of robots (R 21, N 10).

Many participants spontaneously commented on the size of the system. In all groups of participants, some found the system to be too large, whereas a few thought the size to be appropriate for the available space. Some participants criticized the appearance of the lab prototype and wished for a “nicer” and “friendlier” design, possibly with a face. Others, on the contrary, thought it looked “cool” or fit the purpose. Opinions were also divided regarding the velocity of the lab prototype. Nurses positively described the movements as “smooth”, “gentle”, “controlled”, and “cautious”. However, some nurses also felt that the slowness of movement would result in too much time when in use.

The time factor also played a role in the explanation of negatively rated items in the questionnaire. One reason for fearing an equal or higher workload associated with the lab prototype was that the time might be allocated only to operating, picking up and cleaning the lab prototype. A professional nurse explained the concern of negative effects on the staffing ratio in relation to the time factor. She thought that time and efficacy were very important and that is why it is said that one should get by without a colleague (N 15). Concerning the fear that the robot would frighten care recipients, nurses primarily thought of older patients and people with dementia and care recipients who could not classify the lab prototype or generally did not trust technology.

Regarding the perceived usefulness, the nurses particularly saw the greatest added value in the reduction of physical strain, especially on their backs, and in working independently of other colleagues. Those nurses who rated the usefulness more critically in the questionnaire mentioned the effort required to use the lab prototype and thought that they might be faster on their own. Other nurses wished for more application scenarios than the two presented in the live demonstration and therefore a more diverse employment. When asked about an improvement or worsening of the care situation for care recipients, one relative commented that the situation would neither improve nor worsen for care recipients, since the lab prototype is only intended to support nurses (R 30).

## Discussion

This study allowed us to understand and evaluate from multiple perspectives the ethical concerns, perceived usefulness and the intention to use related to the PfleKoRo lab prototype. The use of a questionnaire highlighted the opinions of participants while the open-ended questions provided more detailed information.

Predominantly, the views and perspectives of care recipients, relatives as well as professional nurses were positive in regard to the PfleKoRo lab prototype. The responses of the participants to the survey as well as to the open questions revealed that they had contemplated the benefits of the lab prototype in relation to ethical aspects such as being intimidated by it or fearing that it might endanger the care recipients. Participants also anticipated the indirect impact such a robotic system might have on the structure of the staffing ratio and on the workload for nurses, making it higher or at least not diminishing it. These questions are to be addressed in further research projects, particularly under ethical considerations. Implementations of such systems need to be evaluated comprehensively.

The participants’ perspectives were influenced by the lab prototype’s perceived capabilities and its potential role. Some professional nurses felt that the lab prototype was too limited and would like it to take on more nursing activities. This idea was also given intense consideration in a recent study where a service robot for care of older people was being evaluated. In the mentioned study, older adults as well as formal and informal caregivers wished that the robot in question were able to accomplish more complex tasks [30]. The robotic tasks may differ between the two studies because different systems are being evaluated, but the principal idea remains the same and that is a system that can perform several complex tasks in a modular way. Furthermore, most care recipients who participated in the current study were open to the idea of an assistive robot. Regarding the appearance of the lab prototype, the care recipients of the current study were even more optimistic than the professional nurses and relatives, as none of the care recipients themselves considered the system to be frightening. The difference in these responses between the participant groups might be explained by the fact that the nurses primarily considered the lab prototype would be frightening to people with dementia, who were not included in this study. However, the optimism of the care recipients in the current study agrees with the results of other studies that showed that older adults had a positive response with respect to an assistive robot delivering objects [37] and a robot with abilities like playing cards [22]. Furthermore, in a recent study the authors also discussed the openness to accepting robots and stated that the results of a survey in Germany revealed that 82% of participants aged 60 or older could imagine benefiting from the use of a service robot in their household if it allows them to stay longer in their own houses [38].

The results of the current study showed that 23% of nurses fear an equal or higher workload through robots. In earlier phases of the PfleKoRo project, focus groups were conducted with care recipients, relatives and nurses in order to gather their ethical concerns about the lab prototype. The results regarding the fear of a higher workload for nurses if assisted by the lab prototype were consistent with the results of this study [39]. However, the results of a recent study that evaluated nurses’ views in regard to using robots in pediatric units revealed that robots could reduce nurses’ workload and the utilization of nursing services as well as allow nurses to manage their time better [35]. This disagreement in points of view may be due to the fact that the study by Liang et al. (2019) was assessing points of view regarding robots in general, whereas in this study a lab prototype that performs specific actions was being evaluated.

Furthermore, in an earlier study, caregivers in a nursing home stated that if a robot could assist them with some of their tasks, they would then have more time to spend with the residents [40]. Another study also mentioned that if a robot takes over the repetitive nursing activities that may lead to frustration, then the nurses would be available for the valued interactions with older adults [28]. This idea is in accordance with the results of our paper, since 82% of respondents to the PfleKoRo survey denied fearing a disruption of interaction between nurses and care recipients due to the use of the lab prototype.

82% of the participants in this current study reported being undisturbed about image and data processing through the camera and microphone attached to the lab prototype. This might be partially due to the fact that it was communicated to the participants in the questionnaire that the camera and microphone do not save any care recipients’ information and are not connected to the internet. However, evidence of a recent study showed that its participants had privacy and ethical concerns regarding an exposure of users’ information during robotic use [41]. Similarly, in another study, healthcare professionals were concerned about the use of a camera on a robot as a video surveillance of residents in a retirement village [40]. In our earlier focus groups, when asked about data privacy and the presence of a camera and microphone on the lab prototype, participants gave divergent answers. Some thought that this would not lead to violation of patients’ privacy because the data is not saved. However, others rejected the idea of filming and recording care recipients altogether, even if the data is not being saved.

Moreover, the results of a scoping review showed that health and social care professionals did not feel threatened by robots in the workplace. Instead, they expressed that their job was not affected by the robot and that the robot may have positive effects [42]. These results are in alliance with the results of this study, since 93% of nurses rejected the idea that the lab prototype could lead to a lower appreciation of their work. Professional nurses considered having an assistive system as a benefit instead of rejecting the system because it might be threatening to their job.

With reference to the unfair allocation of the system, only 19% of professional nurses were worried about this. In a recent study, nurses and residents in a retirement home were asked questions about robots. They were also asked to make suggestions. They recommended one robot for each resident [18]. This shows that allocation of robotic systems might prove to be an issue if there are not enough robots to assist when needed.

In addition, 84% of participants did not consider that the lab prototype could be a threat to a care recipient’s safety. The issue of safety when it comes to robots has been mentioned in numerous articles pertaining to robotics in healthcare. In an earlier study, safety issues when developing a system were brought up several times and the importance of a system’s safety was given intense consideration [43]. Three studies about robotics in healthcare expressed how imperative it was for the robot to follow the current safety guidelines including hygiene procedures and the lack of injury risk for care personnel and care recipients [40, 43, 44]. No healthcare institution would ever jeopardize the safety of its care recipients by using insecure systems or systems that do not comply with safety guidelines. Therefore, robotic systems should be tested numerous times before being used on care recipients.

Finally, in this PfleKoRo study, participants were asked to evaluate a lab prototype that does not work independently but only assists nurses in their daily work. The results were mainly positive. These results cannot be generalized for all types of robots since some are built to work independently. In an earlier study, the authors tried to paint a representative picture of the acceptance of assistive robots in nursing by adults in Germany [38]. Based on the results of three surveys, the authors passed on three main messages. Firstly, they revealed that the acceptance of robotic care was dependent on the fact that robotic care did not replace human care but supported it. Secondly, they stated that some participants rejected the whole idea of robots in healthcare on a matter of principle regardless of the robot‘s characteristics. Thirdly, they revealed that of the participants aged 70 or more, those who were women and those with medical or nursing training were more critical of robots [38]. These results show that healthcare professionals and care recipients in Germany are more receptive to robotic care when its main purpose is to support human care.

### Methodological considerations

Initially, in the PfleKoRo project, the plan was to develop a lab prototype that could be tested by professional nurses on real hospital wards by the end of the project. Unfortunately, this plan had to be modified due to the consequences of the SARS-CoV-2-pandemic that led to a delayed development. Thus, there are no results on the usability available so far and direct self-experience by the participants is missing, resulting in an incomplete portrayal of acceptance.

Since not one tool could be found in the literature that was specific to the aspects that the authors wanted to address in this study, the authors themselves developed the survey used here. To check the comprehensibility, the survey and guidelines for the follow-up discussion were pretested with the three professional nurses who were part of the research team. A further limitation of this study is the random sample without a sample size calculation. It can be assumed that the sample of the current study is not representative as the participants were recruited at random from only one university hospital and one nursing home where innovative technologies and research projects are more in use than in other institutions. In addition to the culture of the institutions, technical skepticism, age and gender of the participants, other factors might have influenced the evaluation results, but were not assessed here. No method was used to adjust for non-representativeness of the sample. It should be taken into account that our results refer specifically to the PfleKoRo system and its addressed application scenarios and are therefore not generalizable to other systems.

## Conclusion

This study has revealed various aspects relevant to the acceptance of a robotic system in healthcare from the viewpoints of professional nurses, care recipients and their relatives. The majority of participants in all groups showed a positive and open attitude towards the lab prototype evaluated in this study. However, concerns were frequently raised by both care recipients and relatives regarding the effects of robotic use on the staffing ratios as well as the potential increase in workload for nurses due to the usage of the robotic system. Addressing these concerns is an essential ethical aspect that should be considered.

Bearing in mind that different participant groups focused on specific aspects of the robot, it would be intriguing to conduct separate studies for each group and delve deeper into their individual perspectives on what they consider important. Expanding the research to include a larger population would provide more comprehensive information and enable the generation of results that could be applied to various robotic systems.

Regarding the current state of development, the acceptance among the participants was high, and ethical concerns were relatively minor. Moving forward, it would be beneficial to explore the acceptance in further developmental stages of the system, particularly when usability testing is implemented, which was not possible in the current study.

### Electronic supplementary material

Below is the link to the electronic supplementary material.

Supplementary Material 1

Supplementary Material 2

Supplementary Material 3

Supplementary Material 4

Supplementary Material 5

Supplementary Material 6

Supplementary Material 7

Supplementary Material 8

Supplementary Material 9

Supplementary Material 10

## Data Availability

The datasets generated and analyzed during the current study are not publicly available since the participants did not consent to the publication of the transcripts for all purposes. They are, however, available from the corresponding author on reasonable request.

## Abbreviations

N: Nurse
R: Relative
