# Supplementary figures and images for: Acceptance of a robotic system for nursing care: a cross-sectional survey with professional nurses, care recipients and relatives
Source: BMC Nurs. 2024 Mar 14;23:179. doi: 10.1186/s12912-024-01849-5 (PMC10938668; doi:10.1186/s12912-024-01849-5)

## Results of the care recipients' questionnaires

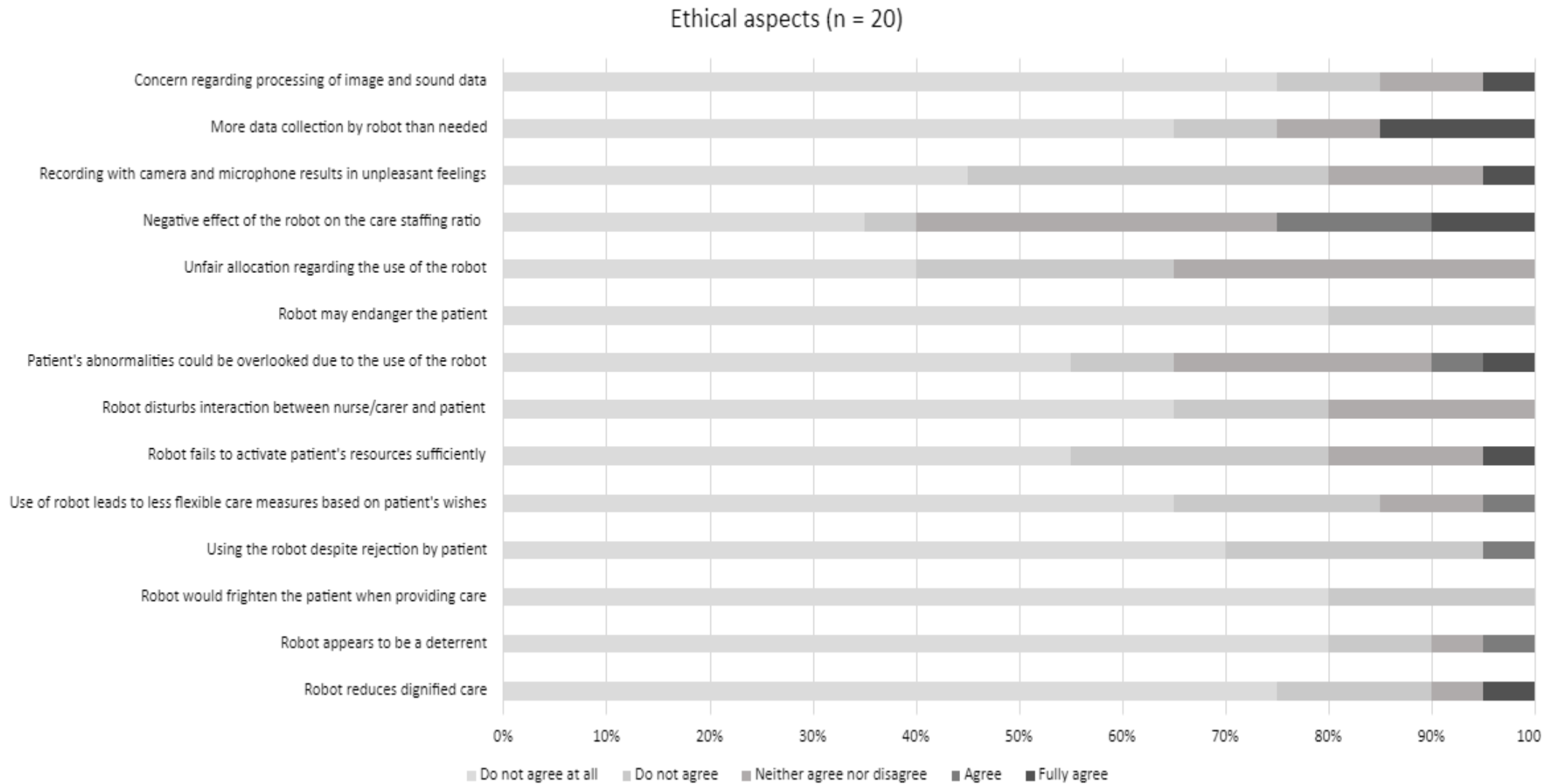

### Usefulness (n = 20)

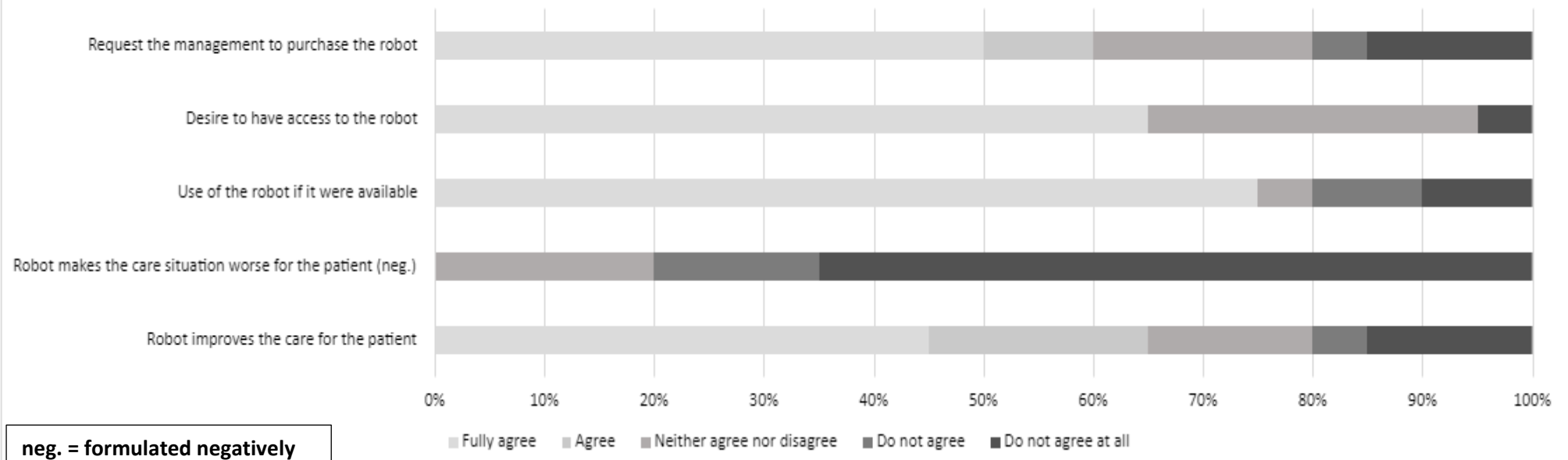

Supplement: Supplementary file 7 — Supplementary Material 7 [file 12912_2024_1849_MOESM7_ESM.pdf]

## Results of the professional nurses' questionnaires

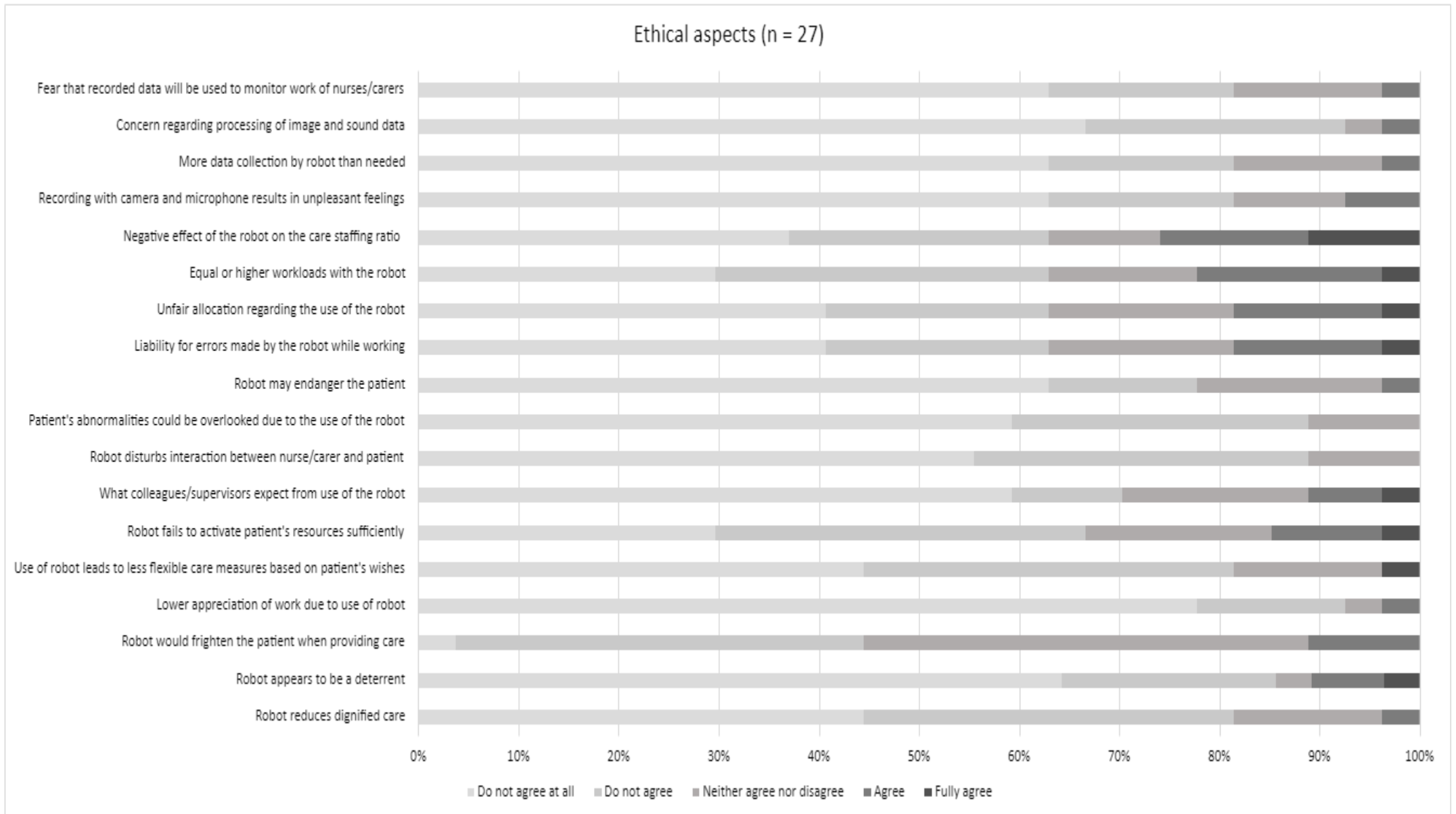

Usefulness (n = 27)

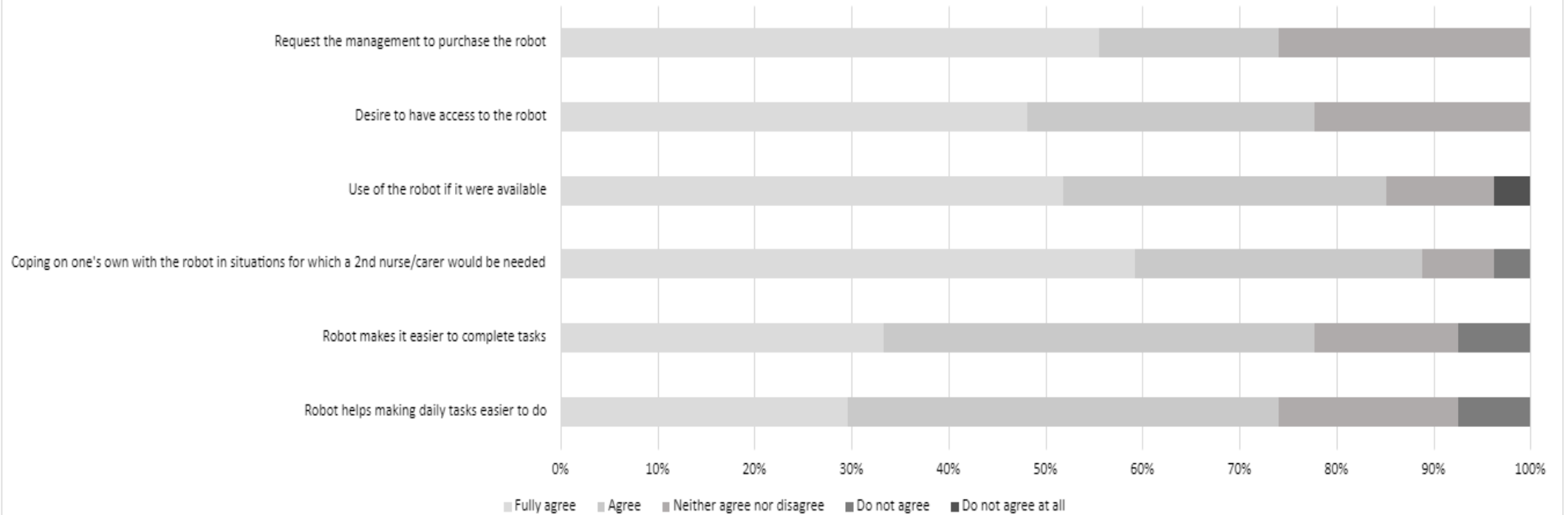

Supplement: Supplementary file 8 — Supplementary Material 8 [file 12912_2024_1849_MOESM8_ESM.pdf]

## Results of the relatives' questionnaires

### Ethical aspects (n = 20)

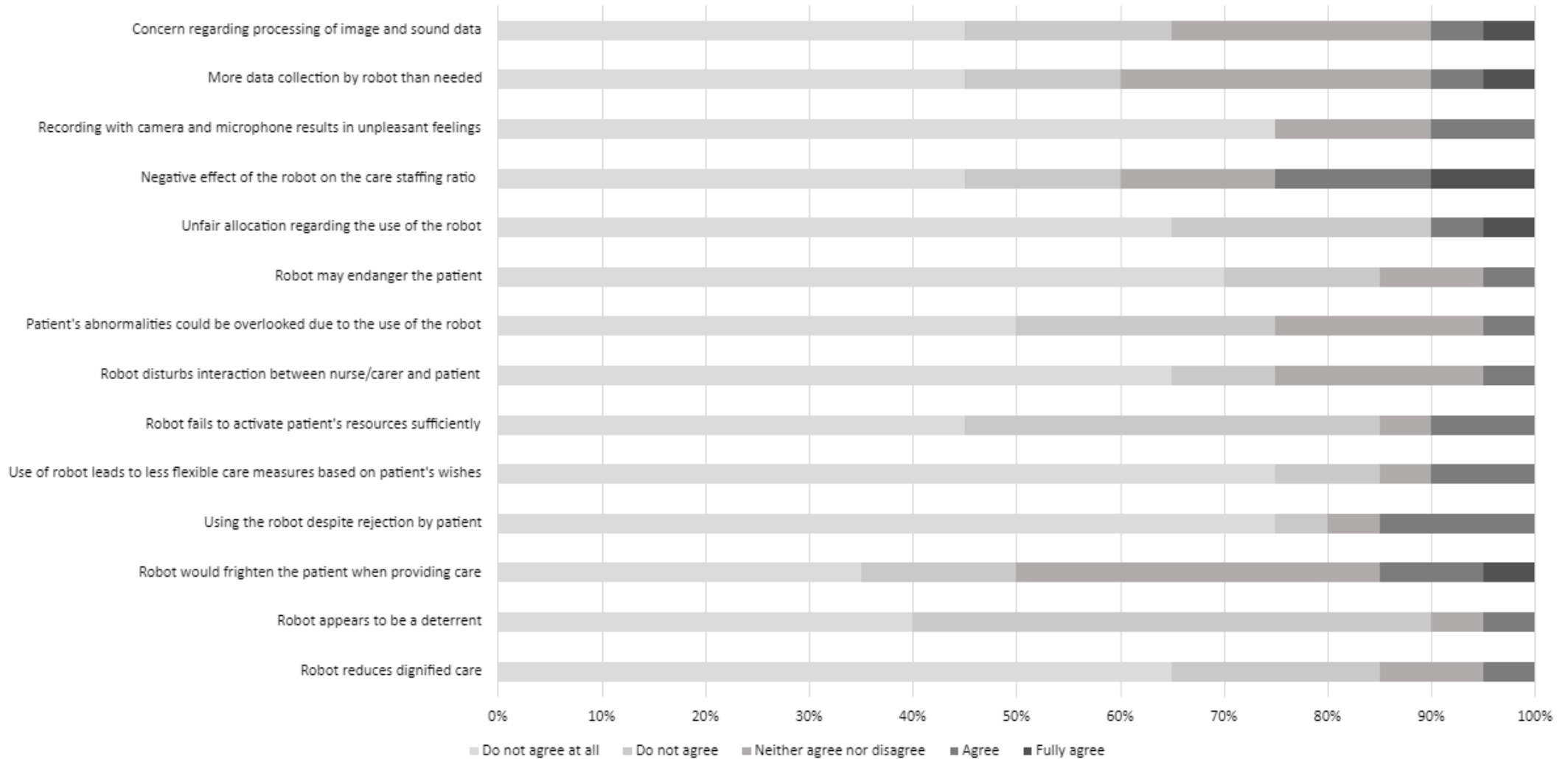

### Usefulness (n = 20)

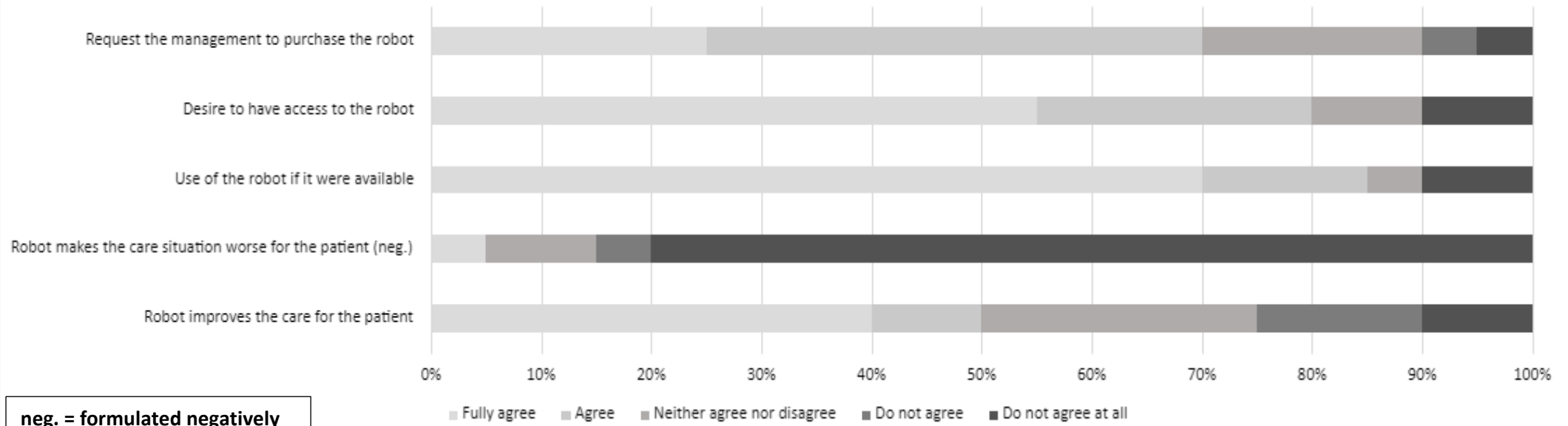

Supplement: Supplementary file 9 — Supplementary Material 9 [file 12912_2024_1849_MOESM9_ESM.pdf]
